# Enabling sensitive and precise detection of ctDNA through somatic copy number aberrations in breast cancer

**DOI:** 10.1038/s41523-025-00739-6

**Published:** 2025-03-08

**Authors:** Riccardo Scandino, Agostina Nardone, Nicola Casiraghi, Francesca Galardi, Mattia Genovese, Dario Romagnoli, Marta Paoli, Chiara Biagioni, Andrea Tonina, Ilenia Migliaccio, Marta Pestrin, Erica Moretti, Luca Malorni, Laura Biganzoli, Matteo Benelli, Alessandro Romanel

**Affiliations:** 1 Department of Cellular, Computational and Integrative Biology, University of Trento, Trento, Italy; 2 Translational Research Unit, Department of Oncology, Hospital of Prato, Azienda USL Toscana Centro, Prato, Italy; 3 Department of Oncology, Hospital of Prato, Azienda USL Toscana Centro, Prato, Italy; 4 Medical Oncology Unit, Azienda Sanitaria Universitaria Giuliano Isontina, Gorizia, Italy; 5 Present Address: Department of Experimental and Clinical Biomedical Sciences, University of Florence, Florence, Italy

**Keywords:** Breast cancer, Translational research, Cancer genomics, Diagnostic markers

## Abstract

Cell-free DNA (cfDNA) extracted from peripheral blood has emerged as a crucial biomarker source in oncology research. To enhance the detection of somatic copy number alterations (SCNAs) and circulating tumor DNA (ctDNA), we developed eSENSES, a 2 Mb breast cancer-targeted NGS panel. It includes 15,000 genome-wide SNPs, 500 focal SNPs in breast cancer driver regions, and exons from 81 commonly altered genes, alongside a custom computational approach. We assessed the performance of eSENSES using both synthetic and clinical samples showing that eSENSES can detect ctDNA levels below 1%, exhibiting high sensitivity and specificity at 2-3% ctDNA levels. In patients with metastatic breast cancer, ctDNA estimations correlated with disease progression. When compared with other technologies and state-of-the-art approaches, eSENSES demonstrated enhanced performance. eSENSES provides a reliable, powerful and cost-effective tool for monitoring disease progression and guiding therapeutic decisions in breast cancer patients.

## Introduction

Breast cancer is a multifaceted disease affecting millions of women worldwide. It is characterized by diverse histological characteristics, metastatic potential and treatment responses. In both early and advanced stages, tissue sampling is essential for pathological assessments and selecting biomarker-based therapies^1^. However, these procedures can delay diagnosis and are often complicated by tumor heterogeneity, sampling limitations, and their invasive nature.

Circulating biomarkers, such as cell-free DNA (cfDNA), are gaining popularity as non-invasive new biomarker source in the field of oncology^2–9^. While early detection of ctDNA signal promises to improve cancer diagnosis^10,11^, the quantification of tumor-derived content in plasma cfDNA (referred to as ctDNA) from cancer patients can be used to monitor the response to therapeutic interventions^12–15^. Indeed, plasma cfDNA carries the genomic characteristics of tumor cell material shed into the bloodstream and in the presence of metastatic disease and/or of multifocal tumors, where single tissue biopsies would fall short in allowing heterogeneity assessment, ctDNA represents an ideal alternative to capture the disease genomic features. Although ctDNA fraction in plasma cfDNA samples ranges typically from 0% to 25%, several studies demonstrated the prognostic value of ctDNA and the ability to track tumor dynamics through the analysis of genomic lesions detected in the circulation of cancer patients^16,17^.

However, biological and technical issues can influence the ability of cfDNA next-generation sequencing (NGS) assays to accurately stratify patients, especially at low ctDNA fractions^18^. Indeed, independently from the NGS approach that is used, low ctDNA fraction strongly influences and limits the detection sensitivity and specificity of different types of genomic events. In particular, somatic copy-number alterations (SCNAs) are challenging to detect and characterize in samples with ctDNA below 20%^19^.

SCNAs are changes in the number of copies of specific genomic regions and their detection and characterization provides information about the progression and aggressiveness of a cancer that are fundamental to guide treatment decisions. Breast cancer is a heterogeneous disease characterized by the presence of both large and focal SCNAs^20^ and different breast cancer subtypes typically exhibit distinct SCNA patterns^21^. Large SCNAs, involving extensive chromosome segments, contribute to genomic instability and resistance to conventional therapies^22^. Focal SCNAs, which affect specific genomic regions, can lead to the overexpression of key oncogenes like *ERBB2* and *CCND1* or the loss of tumor suppressor genes like *TP53* and *PTEN*.

Various methods, including whole-genome sequencing^23^, whole-exome sequencing^23,24^, and targeted sequencing assays^25–27^, have been developed to detect SCNAs from cfDNA and estimate ctDNA levels across different cancers. However, characterizing samples with ctDNA fractions below 10%-15% remains highly challenging, regardless of the method’s advantages and disadvantages. Furthermore, none of the existing approaches can comprehensively characterize both large-scale and focal SCNAs. Hence, although SCNAs hold clinical relevance with important prognostic implications for localized and metastatic breast cancer patients, their detection and characterization through cfDNA NGS non-invasive assays is still hindered and innovative approaches are needed.

In this work, we propose an innovative and cost-effective breast-cancer specific platform that combines a custom NGS cfDNA panel with tailored computational approaches. This panel integrates genome-wide and focal common SNP loci to enhance the sensitivity and specificity of SCNA detection and ctDNA detection and quantification. Additionally, it includes exonic regions of genes frequently mutated in breast cancer, aiding in the interpretation of complex genomic profiles. Ultimately, this technology aims to improve the implementation of liquid biopsies in clinical practice for breast cancer patients.

## Results

### Design and development of a breast cancer panel for the sensitive detection of SCNAs, SNVs and tumor signal from cfDNA

To enhance the detection and quantification of ctDNA and to enable precise characterization of genome-wide and focal somatic copy number aberrations SCNAs in breast cancer patients, we developed a custom targeted sequencing panel, named eSENSES (*e*nabling *S*ensitive and precis*E* detectio*N* of ctDNA through *S*omatic copy number ab*E*rrations in brea*S*t cancer). The panel leverages patients’ genetic backgrounds to improve estimates of allelic imbalances. Because allelic imbalance can only be measured using inherited heterozygous SNPs, our panel design is enriched for genome-wide and gene-specific SNPs with high global minor allele frequency (gMAF). We hypothesized that this approach would maximize the sensitivity of SCNA detection. Our goal was to achieve a genome-wide SNP density of 5 SNPs per megabase (Mb) and, based on previous work^27^, an average of 150 SNPs per focal genomic region containing genes frequently deleted or amplified in breast cancer, including *CCND1*, *ERBB2*, *PTEN*, *TP53*, and *ESR1*.

Additionally, to facilitate parallel and effective profiling of Single Nucleotide Variants (SNVs), we incorporated insights from large-scale breast cancer genomic studies^28–34^. This allowed us to include in our panel the exonic regions of genes that are recurrently aberrant in both localized and metastatic breast cancer (Supplementary Data 1). Overall, our panel comprised approximately 15,000 SNPs and over 2000 exons from 81 genes, spanning a total of around 2.3 Mbp (Supplementary Data 2).

**Fig. 1:**
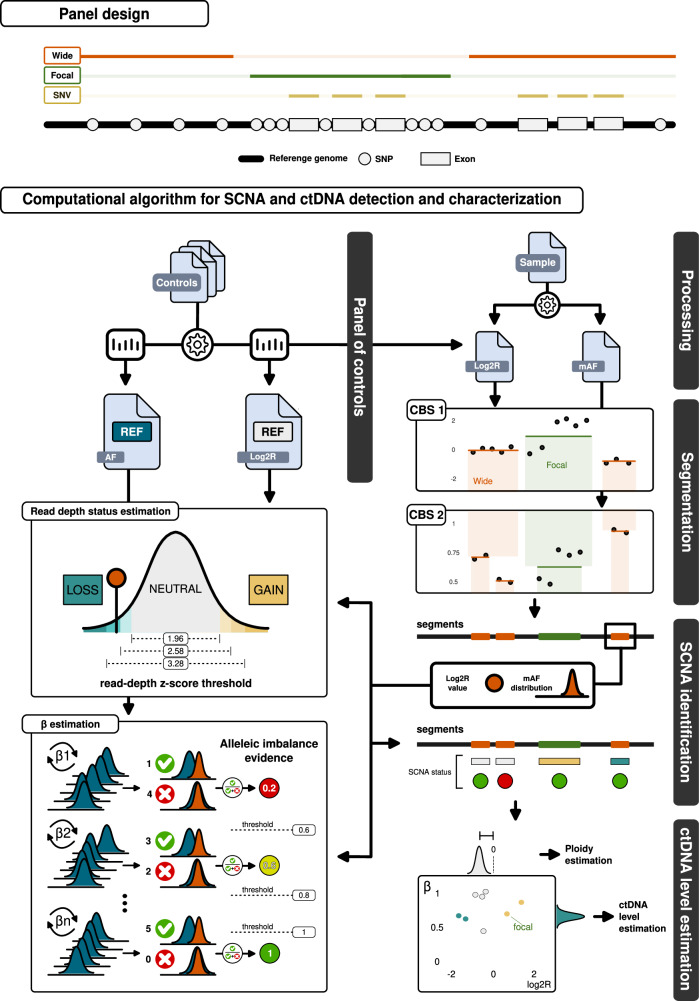
Panel design and eSENSES workflow. The panel design is illustrated at the top of the figure. It includes targeted exons of common breast cancer genes and heterozygous SNPs evenly distributed across the genome (“wide”), as well as SNP-enriched regions specific to certain breast cancer driver genes (“focal”). Additionally, the exons within the wide regions are specifically designed to capture potential SNV events. The panel is paired with a customized computational workflow designed for the sensitive and specific detection of SCNAs and the estimation of ctDNA levels. Control cfDNA samples are processed to generate statistics on SNP allelic fraction (AF) and log2 ratio (Log2R) reference distributions, which are then used to create a panel of controls. For a given patient’s cfDNA sample, this panel computes Log2R values while mirrored allelic fractions (mAF) are processed and filtered. Circular binary segmentation (CBS) is applied to Log2R values, and a second CBS is performed on mAF values for each identified segment region. Focal regions are considered in their entirety and are not segmented. SCNA identification relies heavily on the panel of controls, which is used to estimate the read depth copy number status and its β for each obtained segment—a value proportional to the allelic imbalance of the SNPs in the segment. The sensitivity of these estimations for read depth status and β is adjusted through the parameters of *read-depth z-score* and *allelic imbalance evidence*. The assigned SCNA, along with its respective copy number status, β, and Log2R values, are then used to estimate the ploidy of the sample and the ctDNA level. These values can also be combined to explore the allele-specific (Log2R vs β) SCNA space.

The panel is matched with a tailored computational approach that, exploiting the panel design, enables sensitive and specific detection of somatic copy number alterations and ctDNA detection and estimation also in patients with low ctDNA level. Briefly, we developed a novel SCNA detection algorithm (Fig. 1) that integrates: (i) a read-depth estimation module which models the local and global coverage noise observed across all captured genomic regions and (ii) a SNP-based estimation module that is based on the detection of SNPs’ allelic imbalance. These steps are then followed by estimation of tumor ploidy with data correction, copy number states computation and ctDNA level detection and estimation. The approach embeds the use of a pre-computed panel of controls that provides local and global read-depth and allelic fraction statistics for all captured genomic regions and SNPs and that is generated only once using a set of pre-defined control samples.

For an initial evaluation of eSENSES performance, we conducted plasma sequencing on samples from 20 healthy individuals. We examined the depth of coverage distribution across all captured genomic regions and explored the distribution of allelic fractions for SNPs. Overall, we observed both reasonable read duplication rates (on average 21.6%) and off-target reads (on average 18.1%). Considering processed data, we observed on average a sample’s mean depth of coverage of about 750x (Supplementary Fig. 1) with roughly 80% of the captured genomic regions exhibiting stable relative coverage across the samples (CV < 20%). In line with the panel design, each individual displayed approximately 40% heterozygous SNPs out of all those captured, ranging from a minimum of 38% to a maximum of 44%. Two samples exhibited suboptimal distribution of SNPs’ allelic fraction (Supplementary Fig. 2) and were excluded from further analyses.

### Performance of ctDNA detection and estimation

To assess the detection and estimation limits of our approach and to fine tune the parameters of our algorithm, we employed an *in-silico* approach and built a large benchmarking dataset exploiting the synggen computational tool^35^. Synggen is a software we recently developed for the scalable generation of large-scale realistic and heterogeneous cancer sequencing synthetic datasets, including cfDNA synthetic datasets. Specifically, we utilized sequencing data derived from the plasma samples of the 18 healthy individuals (henceforth referred to as *control* samples) to produce a series of statistical models via synggen. These models encapsulate the distinctive data characteristics inherent to the panel, including coverage distribution across the captured genomic regions and sequencing error frequencies. Subsequently, these models were integrated with germline SNPs and somatic allele-specific SCNA profiles, respectively sourced from 1000 Genomes Project and TCGA datasets, to produce realistic synthetic control and cfDNA samples. These synthetic samples were designed to encompass various levels of ctDNA and average depth of coverage. In particular, average coverage of synthetic samples spanned from ~800x to 2500x and data from 20 representative breast cancer samples from TCGA were used to generate synthetic samples with ctDNA levels spanning from 80% to 0%. Overall, we generated 940 cases/profiles among synthetic cfDNA and control samples.

Our approach’s performance was assessed using the generated benchmarking dataset and various algorithm parameter combinations. The evaluation aimed to gauge and fine-tune eSENSES’s ability to detect tumor signals within synthetic cfDNA samples, known as detection performance, as well as to estimate the ctDNA level. In detail, we started our evaluation generating cfDNA synthetic samples with an average depth of coverage of 800x, representing the average coverage of the data we obtained by sequencing our control samples. Using realistic germline SNP haplotypes and somatic allele-specific SCNA profiles, we generated 260 cfDNA synthetic samples with decreasing ctDNA level, from 80% to 0.1%. Additional 40 synthetic samples with no embedded tumor signal were generated; half (*N* = 20) representing control samples to create the panel of controls and half representing cfDNA samples with ctDNA level at 0% to measure the false positive rate (FPR) of our approach. The performance of our approach was tested for different combinations of *allelic imbalance evidence* (from 0.2 to 1) and *read-depth z-score* (from 1.96 to 3.89) thresholds, representing the key parameters of our SCNA detection algorithm.

**Fig. 2:**
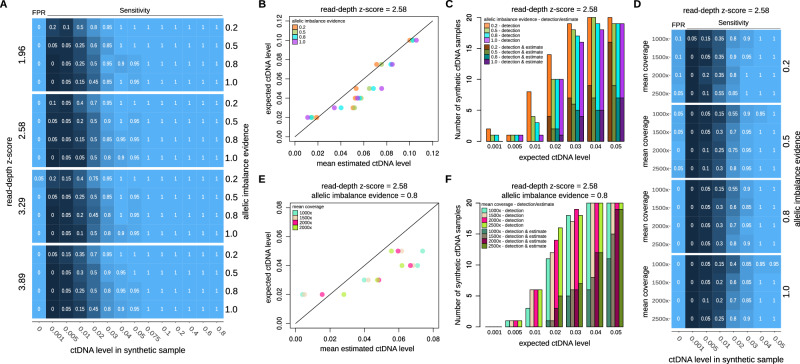
eSENSES performances from the in-silico benchmarking. **A** Sensitivity and FPR results of ctDNA detection for increasing simulated ctDNA level across different read-depth z-score and allelic imbalance evidence parameters’ values. **B** Concordance between the mean estimated ctDNA level, calculated across the results obtained for the simulated cfDNA samples, and the expected ctDNA level. Results are shown for read-depth z-score parameter equal to 2.58 and increasing allelic imbalance evidence parameter’ values. **C** Fraction of samples with ctDNA detection and ctDNA detection and ctDNA level estimation for expected ctDNA levels up to 5%. Results are shown for read-depth z-score parameter equal to 2.58 and increasing allelic imbalance evidence parameter’ values. **D** Sensitivity and FPR results of ctDNA detection for increasing simulated depth of coverage. Results are shown for read-depth z-score parameter equal to 2.58 and increasing allelic imbalance evidence parameter’ values. **E** Concordance between the mean estimated ctDNA level, calculated across the results obtained for the simulated cfDNA samples, and the expected ctDNA level. Results are shown for read-depth z-score parameter equal to 2.58 and allelic imbalance evidence equal to 0.8. **F** Fraction of samples with ctDNA detection and ctDNA detection and ctDNA level estimation for expected ctDNA levels up to 5%. Results are shown for read-depth z-score parameter equal to 2.58 and allelic imbalance evidence equal to 0.8.

As shown in Fig. 2A, no major detection performance deviations were observed across the different parameters’ combinations. As expected, increasing the *read-depth z-score* might slightly reduce sensitivity, while decreasing *allelic imbalance evidence* might increase the False Positive Rate (FPR). Overall, we noted a sensitivity nearing 100% for ctDNA levels as low as 4%-5%, with a slight decline to 80–90% when ctDNA levels reach 3%, and a rapid decrease for levels below 3%. Although supported by low sensitivity, our data suggest that our ctDNA detection limit is as lows as 0.5%. When assessing ctDNA estimation performance (Fig. 2B and Supplementary Fig. 3), we consistently observed a strong correlation with expected ctDNA levels (on average >99%). Of note, for synthetic cfDNA samples with expected ctDNA levels below or equal to 5%, less than 50% of the samples with positive ctDNA detection had also estimations available (Fig. 2C); in addition, no ctDNA level estimations were available for samples with expected ctDNA level below 2%.

Next, we tested to what extent an increase in average depth of coverage would increase the performance of our approach. As illustrated in Fig. 2D and Supplementary Fig. 4, the *in-silico* benchmarking indicates that increasing the coverage could potentially improve sensitivity of ctDNA detection to over 80% even for samples with an expected ctDNA level of 2%. However, achieving this would theoretically necessitate tripling the coverage. Similarly, as shown in Fig. 2E, F and Supplementary Fig. 4, increasing the coverage would also increase the fraction of samples with ctDNA level estimation available. Nonetheless, it would still not enable estimations in samples with ctDNA levels below 2%.

Taking into account the outcomes of our benchmarking analysis, we opted for setting the *read-depth z-score* to 2.58 and the *allelic imbalance evidence* to 0.8. This configuration strikes a balanced compromise between sensitivity and specificity, optimizing performance in both ctDNA detection and ctDNA level estimation.

### Clinical application of the panel in metastatic breast cancer serial samples

To assess the clinical applicability of eSENSES, we profiled 44 cfDNA samples from a cohort of 15 patients with metastatic breast cancer (Supplementary Data 3), collected in the context of a larger collaborative project named MIMESIS^36,37^. For all but one of the patients, we had available plasma samples before the start of the therapy (T0), at the second cycle of therapy (T1) and at progression disease (T2). Two patients had ER + /HER2+ disease, 11 ER + /HER2-, one ER-/HER+ and another one ER-/HER2-.

Sequencing was performed resembling the characteristics of our control samples. In particular, in the generated data, we consistently observed reasonable read duplication rates (on average 20.3%), reasonable off-target reads (on average 19.1%) and an average sample mean depth of coverage of approximately 800x (Supplementary Fig. 5).

**Fig. 3:**
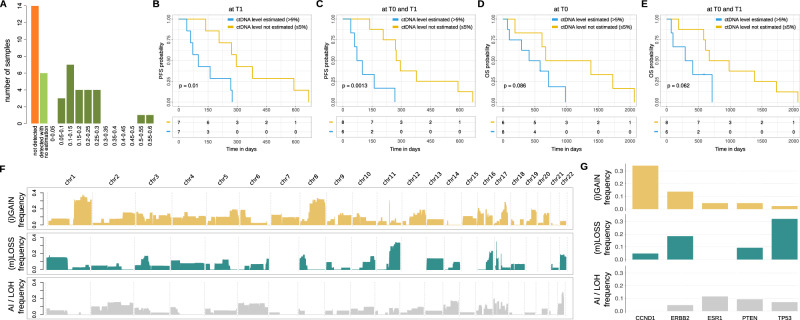
Profiling of the clinical cohort. **A** Distribution of ctDNA detection and ctDNA level estimation across all cohort’ samples. **B** PFS results stratifying samples based on availability of ctDNA level estimation at T1. **C** PFS results stratifying samples based on availability of ctDNA level estimation at T0 or T1. **D** OS results stratifying samples based on availability of ctDNA level estimation at T0. **E** OS results stratifying samples based on availability of ctDNA level estimation at T0 or T1. **F** Landscape of genome-wide SCNAs detected across all cohort’s samples. **G** Landscape of focal SCNAs detected across all cohort’s samples.

For 14 samples across 8 patients, eSENSES was unable to detect any tumor signal. Among the remaining 30 samples with detectable ctDNA, 24 had ctDNA estimations available, with values ranging from 5% to 60% (mean 20.4%) (Fig. 3A and Supplementary Data 4). These values align with the aggressive features of the analyzed patients, showing no significant distribution differences between samples from ER + /HER2+ and ER + /HER- patients (*P* = 0.18). Although not statistically significant, we observed a general decrease in median ctDNA levels from 21% at T0 to 14% at T1. In addition, an increased number of involved sites was observed in patients with detectable ctDNA at T0 compared to those with non-detectable ctDNA (Supplementary Data 5).

Despite the cohort being limited to 15 patients, which reduced its statistical power for clinical analyses, we found that ctDNA estimation served as a prognostic indicator. Specifically, patients with estimated ctDNA levels at T1 had inferior progression-free survival (PFS) (*P* = 0.01, Fig. 3B). The prognostic signal was even stronger when considering available ctDNA level estimations at both T0 and T1 (*P* = 0.0013, Fig. 3C). Additionally, while not statistically significant, patients with estimated ctDNA levels at T0 or both at T0 and T1 exhibited a clear trend toward inferior overall survival (OS) (Fig. 3D, E).

Although assigning precise copy number status to genomic segments at low ctDNA levels is challenging, the somatic copy number aberration profiles (Supplementary Data 6) reconstructed by our approach were consistent with previously published data^29–34^ (Supplementary Fig. 6), showing a clear enrichment of large gains/amplifications in the 1q, 8q, 16p and 17q arms, and significant large deletions in the 8p, 11q, 16q, 17p, and 22q arms (Fig. 3F). Additionally, focal somatic copy number aberration analysis detected, as expected, amplification signal for *CCND1* gene and deletion signal for TP53 gene large fractions of patients (about 35% and 30%, respectively) and a clear amplification signal for *ERBB2* gene in both HER2+ patients for which we had detectable ctDNA (Fig. 3G and Supplementary Fig. 7).

Regarding the analysis of single nucleotide variants, performed exploiting our tool ABEMUS^38^ along with a custom post-processing pipeline, we were able to detect 80 non-synonymous SNVs across 32 genomic positions, with an average of 6 SNVs (from 1 to 20) per patient (across T0, T1 and T2) and an average of 3 SNVs (from 1 to 7) per sample. Median observed allelic fraction of non-synonymous SNVs was 3.7% (from 0.2% to 56%). The complete list of the non-synonymous SNVs detected in all samples is reported in Supplementary Data 7. The most frequently mutated genes at T0 or T2 were *ESR1* (5/15, 33%), *PIK3CA* (5/15, 33%) and *TP53* (4/15, 27%), aligning with published large genomic studies (Supplementary Data 8). *ESR1* mutations were often observed at T0 but not at T2 (Supplementary Fig. 8) and were primarily subclonal (Supplementary Fig. 9), whereas *PIK3CA* mutations were predominantly clonal and detected at both T0 and T2. In some samples where ctDNA was undetectable via SCNA, we identified damaging non-synonymous SNVs in key driver genes, aiding in the interpretation of circulating tumor signals. For example, although patient P03 had a positive ctDNA estimation only for sample T1 (at 7.5%), the *PIK3CA p.E545K* hotspot gain-of-function mutation was detected as heterozygous mutation in all three samples, with allelic fractions of 2.3%, 4.6%, and 1.6% in samples T0, T1, and T2, respectively.

**Fig. 4:**
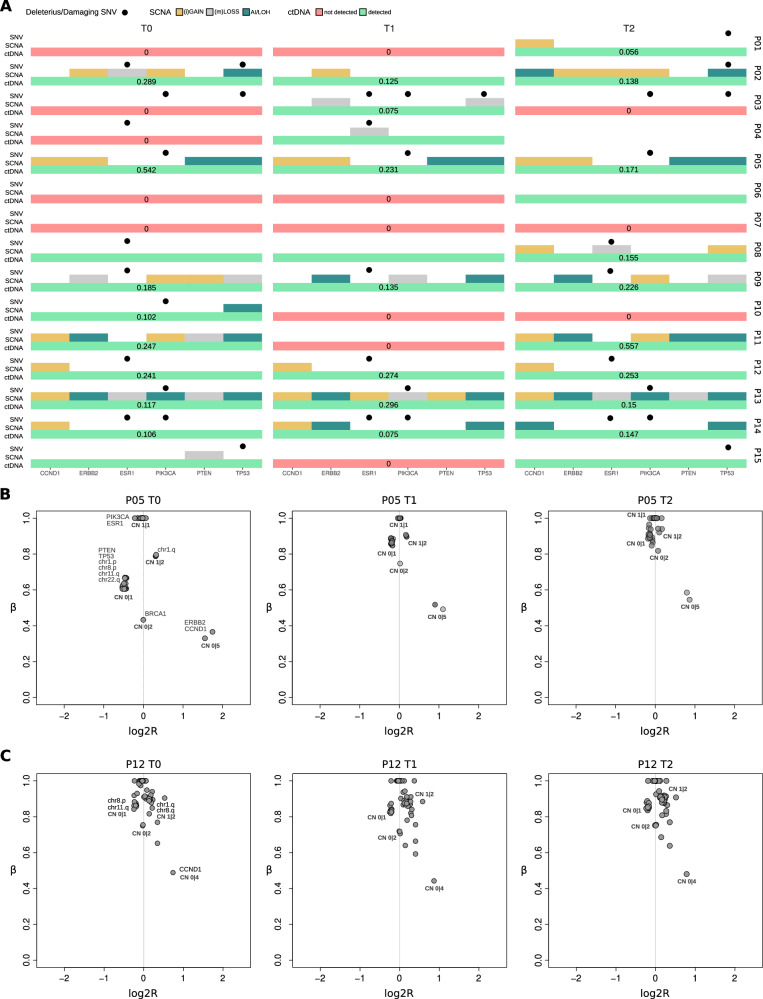
Longitudinal analysis of the clinical cohort. **A** Distribution of SCNAs, SNVs and ctDNA detection/estimation for breast cancer driver genes across all patients at the different time points. **B** Allele-specific analysis of SCNAs across patient’s P05 T0, T1 and T2 samples. **C** Allele-specific analysis of SCNAs across patient’s P12 T0, T1 and T2 samples. Points indicate SCNA segments in the sample and labels indicate genomic position and (in bold) the allele-specific copy number. Points indicate SCNA segments in the sample and labels indicate genomic position and (in bold) the allele-specific copy number.

Overall, the dynamics of arm-level SCNAs, along with focal SCNAs and non-synonymous SNVs in breast cancer driver genes across longitudinal samples, revealed a high genomic similarity of tumor clones between T0 and T2 for the majority of patients (Fig. 4A). In particular, for matched samples with ctDNA estimations above 10%, these similarities can be further explored using a detailed allele-specific SCNA analysis, which leverages the allelic imbalance information available from our approach. For example, the analysis of patient P05 samples shows genomic profiles that are identical across all longitudinal samples, with mono-allelic loss of *PTEN* and *TP53* genes, together with broad mono-allelic losses in p-arms of chromosomes 1 and 8 and q-arms of chromosomes 11 and 22, gain of q-arm of chromosome 1, loss of heterozygosity (LOH) of BRCA1 gene and amplifications of *ERBB2* and *CCND1* genes (Fig. 4B). Another example is depicted in Fig. 4C, where the analysis of patient P12’s samples consistently shows mono-allelic losses in chr8.p and chr11.q, as well as gains in chr1.q and chr8.q, with unaltered ctDNA levels across all time points.

Overall, eSENSES demonstrated prognostic ctDNA level estimations and the capacity to offer sensitive genomic profiling across various abstraction levels. It fully leveraged allelic-imbalance information, while also illustrating the specific temporal evolution of each patient’s cancer.

### Validation of eSENSES performances using independent and complementary approaches

To further validate our approach, we exploited additional profiling data available for our cohort’s patients across the same time points in the context of the collaborative MIMESIS project^37^. More specifically, the same cfDNA samples were also characterized generating low-pass methylation whole-genome bisulfite sequencing (lpWGBS) and analyzed with ichorCNA^23,39^, a state-of-the-art tool for cfDNA WGS data analysis. Given our findings in^37^, which showed that bisulfite pretreatment for WGS does not introduce significant biases in SCNA characterization or ctDNA detection, we were able to directly compare the performance of our approach with a complementary methylation-based assay.

IchorCNA was executed on lpWGBS data using two different configurations suggested by the authors: a *default* run with parameters optimized for estimating ctDNA levels in the context of moderate to high expected ctDNA, and a *sensitive* run designed for estimating ctDNA levels when low expected ctDNA is anticipated.

**Fig. 5:**
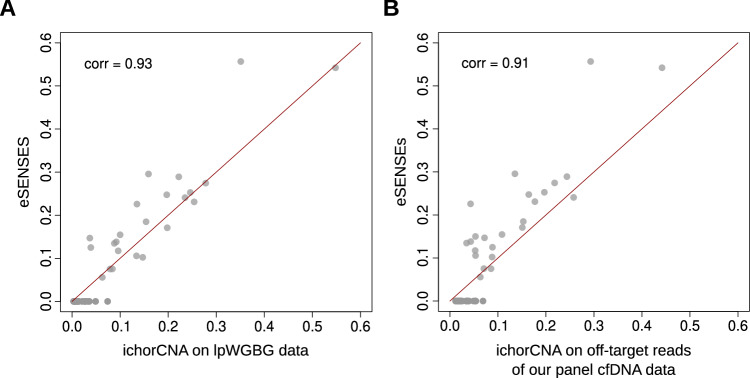
Validation analysis. **A** Concordance of eSENSES ctDNA level estimations with estimations obtained with ichorCNA from lpWGBS samples available for the same patients at the same time points. **B** Concordance of eSENSES ctDNA level estimations with estimations obtained from ulpWGS built from eSENSES off-target sequencing reads.

A high concordance was observed when comparing ctDNA level estimations obtained using the two complementary strategies. Specifically, there was an 80% correlation with the *default* run, which had a clear detection limit of 10% ctDNA (Supplementary Fig. 10). This correlation increased to 93% with the *sensitive* run (Fig. 5A). In this mode, ichorCNA did not provide any negative calls, detecting positive ctDNA levels below 3% in 16 samples, which is the theoretical limit of detection declared by the authors of ichorCNA. Interestingly, considering that the majority of these 16 calls are likely to be false positives, for 11 of them (70%) our approach detected no tumor signal and for the 5 remaining ones our approach was able to detect the presence of ctDNA without however providing a ctDNA level estimation.

To further validate ctDNA of eSENSES and considering that ichorCNA was developed for ultra-low-pass whole-genome sequencing (ulpWGS) data, we utilized off-target reads from our cfDNA samples to achieve an average whole-genome depth of coverage of around 0.2x per sample, thereby emulating ulpWGS data.

On these samples, we first ran ichorCNA in *sensitive* mode, focusing on patients’ data and using control samples to create an ichorCNA panel of normals (PoN). As shown in Fig. 5B, ctDNA level estimation on patients’ cfDNA data exhibited strong concordance between our approach and ichorCNA. This concordance closely resembled the results obtained with lpWGBS data. Also in this case, ichorCNA did not provide any negative calls, detecting positive ctDNA levels below 3% in 11 samples. Among these, 9 had no call by our approach, and the remaining 2 had a positive call with no estimation available, overall suggesting that our approach may offer improved precision in detecting ctDNA signals below the 3% threshold.

To support this observation and further validate the specificity of our approach, we finally conducted a leave-one-out (LOO) cross-validation analysis. For each control sample, we tested the eSENSES estimation using the remaining control samples to construct the panel of controls. Notably, the LOO analysis yielded negative ctDNA results for all control samples, confirming the high specificity of our method.

Overall, these results clearly demonstrate that eSENSES matches the sensitivity of state-of-the-art approaches while maintaining high specificity, thereby preventing the generation of false positive ctDNA calls.

## Discussion

In this work, we introduced eSENSES, a custom targeted sequencing cfDNA panel combined with a tailored computational approach for a sensitive and specific detection and quantification of ctDNA and somatic copy number aberrations in patients with metastatic breast cancer.

By leveraging a comprehensive set of focal and genome-wide SNPs, eSENSES enhances the characterization of SCNAs and their allelic imbalances, providing an advanced framework for analyzing allele-specific SCNAs in low ctDNA breast cancer samples. Additionally, by including exonic regions of recurrently mutated breast cancer driver genes, eSENSES enables the detection and characterization of driver breast cancer SNVs, further improving the understanding and interpretation of complex allele-specific SCNA patterns.

Current NGS methods for estimating the tumor fraction in cfDNA based on SCNA profiles typically utilize either a *horizontal* approach, such as whole-genome^18,39^ or whole-exome sequencing^23,24^, or a *vertical* approach with targeted sequencing^25,26^. While those approaches optimize either sequencing breadth or sequencing depth, eSENSES has the advantage of maximizing both, offering an innovative method that combines the strengths of *horizontal* and *vertical* sequencing. An approach in this direction was proposed for prostate cancer in^40^, where only a thousand genome-wide SNPs and the exonic regions of only 13 genes were captured. Although the authors reported a ctDNA detection limit of 5%, which is one-fold higher than our in-silico estimate for eSENSES, they did not thoroughly explore the sensitivity and specificity of their detection limit.

To enhance the sensitivity of SCNA detection, other methods fully exploiting SNP allelic imbalance have been proposed^27,41^. Specifically, in ref.^41^, an approach was developed to improve SCNA detection in pancreatic cancer cfDNA samples with low ctDNA. Although detection at ctDNA levels <5% was shown to be feasible, the sensitivity remained limited, only large-scale SCNAs were potentially detectable, and deep WES (>200x average depth of coverage) was required to achieve the enhanced performance levels.

Another advantage of eSENSES is that it does not require matched control samples. Instead, it utilizes a pooled set of control samples to build a reference model a single time, which is then applied by our computational approach to analyze all patients’ cfDNA samples.

Our in-silico benchmarking analysis demonstrated that eSENSES, when applied to samples with an average depth of coverage of 800x—resulting in a sequencing cost comparable to that of low-pass whole genome sequencing (approximately 30-40 million reads)—achieves high sensitivity and specificity in detecting and estimating ctDNA levels. This performance is particularly notable in samples with low ctDNA content. Our approach demonstrated the potential to achieve a detection sensitivity of 90% for ctDNA levels as low as 3% with zero FPR and a detection limit at 0.5% ctDNA. Increasing the depth of coverage to approximately 2500x allowed us to maintain a detection sensitivity of over 80% for ctDNA levels as low as 2%, while still ensuring a zero FPR.

The effectiveness of eSENSES was demonstrated on a small cohort of patients with metastatic breast cancer. In particular, the analysis of 44 cfDNA samples from 15 patients demonstrated that ctDNA levels were consistent with aggressive disease features and were associated with both overall survival and progression-free survival. Although our cohort predominantly included ER+ breast cancer patients, with approximately 60% evaluated at the onset of metastatic treatment, the distribution of ctDNA estimates closely mirrored those reported in ref.^42^. This study, focusing on metastatic TNBC patients, found that 70% of analyzed samples had confidently detectable ctDNA, with 63% of patients presenting at least one sample where ctDNA levels exceeded 10%. Notably, this threshold, which aligns with the one used in our study, was also deemed by the authors as adequate for high-confidence SCNA characterization.

The SCNA profiles of the cohort were consistent with established breast cancer profiles described by large genomic studies^28–34^, identifying significant large-scale and focal amplifications and deletions in key genes and genomic regions. Of note, eSENSES was able to precisely delineate the amplification status of *ERBB2* gene’s genomic region in all HER2+ patients with detectable ctDNA and to provide a confident allele-specific characterization of all detected SCNAs when ctDNA levels exceeded 10%. This capability facilitated the precise characterization of intricate SCNA patterns and LOH events.

Furthermore, the SNV analysis accurately identified known non-synonymous point mutations in well-established breast cancer driver genes like *ESR1*, *PIK3CA*, and *TP53*. The patterns in SNV allelic fractions also contributed to the analysis of longitudinal samples from patients, revealing, in conjunction with SCNA profiles, a prevalent high genomic similarity among tumor clones circulating across various disease time points within our cohort.

To further validate eSENSES’s performance, we demonstrated that ctDNA detection and level estimations from our cohort samples closely matched those provided by the state-of-the-art tool ichorCNA. We applied ichorCNA to low-pass WGBS data from the same patients and utilized off-target reads from our targeted approach to simulate ultra-low-pass WGS samples. Importantly, eSENSES exhibited comparable ctDNA detection sensitivity to ichorCNA while maintaining high specificity.

To conclude, we introduced eSENSES, an approach that exploits a combination of focal and genome-wide SNPs with a tailored computational approach, providing a sensitive and specific cost-effective technology for detecting ctDNA and characterizing SCNAs in breast cancer patients. The eSENSES panel demonstrates significant clinical validity in monitoring disease progression and offers a reliable alternative to existing methods, maintaining high specificity and sensitivity.

## Methods

### Selection of SNPs to include in the panel

Two sets of high gMAF SNPs were included in the panel; a first set, including loci distributed uniformly along the human genome to ensure the detection of SCNAs involving extensive genomic regions, and a second set, consisting of SNPs located across genes frequently and focally altered by SCNAs.

For the first set, to ensure greater experimental efficiency, priority in selection was assigned to SNPs included in the Infinium Omni2.5-8 Kit microarray and characterized by a MAF ≥ 0.45, as reported in the dbSNP version v151 catalog^43^ considering all populations included in the 1000 Genomes Project^44^. More specifically, for each chromosomal band, a number of SNPs was selected to ensure a total density of 5 SNPs/Mb, a minimum number of 20 loci for the least populated chromosomal arm (chr21p) and maintaining a spacing of at least 200 bases between two consecutive SNPs.

For the second set, SNPs with gMAF ≥0.35 were selected in genomic windows of approximately 500 kbp both upstream and downstream of 5 selected genes (*CCND1*, *ERBB2*, *PTEN*, *TP53*, *ESR1*) that are frequently affected by focal SCNAs.

In total, 17,012 SNP loci were selected (Supplementary Data 2).

### Selection of genes to include in the panel for SNV analysis

Publicly available data from the cBioPortal for Cancer Genomics^45,46^ and the Integrative Onco Genomics (IntOgen) database^47^ were utilized to define a list of genes of interest in the clinical context of breast carcinoma. Specifically, mutational data from the METABRIC studies^28–30^, TCGA PanCancer Atlas (www.cancer.gov/tcga) INSERM^31^, MSK-IMPACT^32^, and The Metastatic Breast Cancer Project (www.mbcproject.org) were analyzed to associate a mutation frequency with each gene. A gene was considered mutated if at least one of its coding regions was found altered by the presence of at least one somatic point mutation causing a change of a base to a different and “non-synonymous” one.In total, 17,012 SNP loci were selected (Supplementary Data 2).

To ensure the selection is as accurate and informative as possible, the data and their mutational frequencies were stratified based on the molecular subtype of the tumor (HER2 + , HR + , Triple Negative) and its stage of evolution (primary or metastatic). This stratification allowed the selection of the most frequently mutated genes (*N* = 25) for each of the 6 classes. The unique set of genes identified from this initial selection (*N* = 68) was validated and further extended by analyzing tumor-specific aberration frequencies reported in the IntOgen database. The combined results of these two analyses identified 81 genes of interest, whose coding regions (exons, excluding UTRs) were selected for sequencing. The final selection included 2149 genomic regions, covering the exons of 81 genes (Supplementary Data 1 and Supplementary Data 2).

### Panel design

All selected SNPs and coding regions of genes of interest were included in a single BED file. This file served as input for the Illumina HyperDesign software (hyperdesign.com), with stringency parameters (a value defining the specificity of a probe for the target region; min = 1, max = 20, optimal interval for unique probes from 1 to 4) and overhang (a value defining the number of bases by which the probe deviates from the target region; min = 0, max = 120, optimal interval from 0 to 30) set to 4 and 30, respectively. The output produced by HyperDesign defined the optimal genomic coordinates for 17,040 sequencing probes, covering a total of approximately 2.3 Mbp and including 14,829 SNPs (around 90% of the initially selected SNPs) and 2075 genes’ exonic regions (about 95% of the initially selected ones).

### Samples and patients’ characteristics

Plasma samples (*n* = 44) were collected from 15 patients with metastatic breast cancer. Clinical and pathological characteristics for each of the patients are reported in Supplementary Data 3 and Supplementary Data 5. In particular, samples were drowned at baseline (T0, *n* = 15), after one cycle of treatment (T1, *n* = 15) and at disease progression (T2, *n* = 14). Plasma samples were prepared within an hour from blood collection and stored at −80 °C until cfDNA extraction. Normal K2EDTA plasma samples from 20 female healthy donors were purchased from Precision for Medicine (Precision for Medicine, MA).

### cfDNA extraction, library preparation and sequencing

Plasma cfDNA was isolated by QIAmp Circulating Nucleic Acid Kit (Qiagen) and quantified by Qubit (Qubit dsDNA High Sensitivity Kit, Life Technologies). Libraries were prepared starting from 20 ng of DNA using KAPA HyperPrep Kit and Kapa Universal UMI adapter (Roche), according to the manufacturer’s instructions. Libraries size distribution and concentration were analyzed respectively by Bioanalyzer DNA High Sensitivity Kit (Agilent) and Qubit (Qubit dsDNA High Sensitivity Kit, Life Technologies) before target enrichment with the 17,040 customized Kapa hyper Cap Target Enrichment Probes (Roche). For the enrichment step four samples were combined using 500 ng of each amplified indexed library. The enriched DNA samples were amplified according to the manufacturer’s instructions with 16 cycles of Post-Capture PCR. The amplified enriched DNA samples libraries were quantified by Qubit dsDNA High Sensitivity Kit and libraries size distribution were evaluated by Bioanalyzer DNA High Sensitivity Kit. Sequencing was performed on Illumina Novaseq 6000 generating 150 bp paired-end reads.

### Sequencing data pre-processing

Reads were trimmed to remove adapters and UMI extraction, consensus and sorting were performed using UMI_tools (version 1.1.2)^48^, fgbio (version 2.0.2), and GATK (version 4.2)^49^. Alignment to the human GRCh38 reference genome was performed using BWA-MEM. Realignment and recalibration were performed using GATK. MD tags were calculated using samtools calmd (version 1.7)^50^ and overlapping read pairs were clipped using bamUtil (1.0.14). PaCBAM was used to generate pileup and depth of coverage statistics’ files^51^. In particular, we used *rc* files, reporting the average depth of coverage of all captured genomic regions along with their GC content, and *snps* files, reporting pileup statistics for all considered SNPs such as total coverage, coverage supporting the reference allele, coverage supporting alternative allele, and the variant allelic fraction (AF).

### Reference mapping bias correction

Reference Mapping Bias (RMB), namely the presence of a main AF peak value different from the expected 0.5, is addressed to ensure proper downstream analysis of SNPs’ AF data and comparison of AF distributions from independent samples, similarly to what previously described in ref.^52^. The peak correction was applied separately for all cfDNA samples, applying a Kernel Density Estimation on the heterozygous SNPs AF distribution and extracting peaks by computing the local maxima of the smoothed distribution^27^; the central peak was extracted and data centered to the 0.5 theoretical value.

### Panel of controls

Sequencing data of control samples was used to generate a panel of controls, which characterizes the captured genomic regions of all genes of interest and all SNPs.

First, read depth of coverage of all captured regions across all control samples was normalized for both GC content and sample’s mean coverage. Normalized read depth (RD) was then used to compute, for each region across all control samples, mean and standard deviation values. Then, we performed a leave-one-out procedure in which, for each control sample *i* and each captured region *c*, a log2 ratio (log2R) value was calculated using the following formula:

1 $${\log 2R}_{{ci}}=\log 2R\left(\frac{{{cov}}_{{ci}}}{{mean}({{cov}}_{{cn}-i})}\right)$$

with ${{cov}}_{{ci}}$ being the RD of the control sample *i* in region *c* divided by the mean of the RD of region *c* across all control samples excluding sample *i (*${{cov}}_{{cn}-i}$*)*. In this way a table of log2R values for each captured region across each control sample was obtained. While performing this operation, a *mask ratio* parameter (set at 0.5) was used. This value determines the minimum percentage of samples having finite values for a considered captured region in order to perform the log2R calculation. If the *mask ratio* was not reached that region was not included in the panel of controls. Overall, read depth of coverage statistics are organized in two tables: the first, referred to as *rc table*, having the RD mean and standard deviation for each captured region across all controls; the second table, referred to as *log2R table*, having for each captured region all the controls’ log2R values.

Then, for SNPs’ AF data two main data structures were built: (1) a collection of SNPs summary statistics; (2) AF dispersion stratified by changes in SNPs local coverages. Briefly, for each control sample, captured SNPs that have a heterozygous genotype (0.2 < AF < 0.8) were kept. Summary statistics for each heterozygous SNP across all control samples were computed, including, AF distribution mean, coefficient of variation, proportion of samples out of N harboring the heterozygous genotype, and mean coverage. AF dispersion was instead modeled collecting AF standard deviations stratified by local coverage quantiles Q (min 0%, max 100%, step 10%).

### SCNA identification

For a given cfDNA plasma sample, raw read depth data was processed as mentioned above. Upon calculation of log2R for all captured regions, Circular Binary Segmentation (CBS)^53^ was performed, using the R package DNAcopy (version 1.68)^54^, to identify putative read depth distribution change points representing copy number variations. The segmentation analysis was performed considering single arms of each chromosome separately. Focal genes’ regions, enriched for high MAF SNPs in our panel, were smoothed in order to prevent over-segmentation, in other words, segmentation was not performed on these genes and their SNPs enriched regions were considered in their entirety for gaining a better signal.

Then, for each identified segment, a second run of CBS was performed. This time upon mirrored AF values (mAF), calculated as:

2 $${mAF}=\,\left\{\begin{array}{ll}1-{{SNP}}_{{af}}, & {{SNP}}_{{af}} \,>\, 0.5\\ \quad \,\,\,\,{{SNP}}_{{af}}, & {otherwise}\end{array}\right.$$

mAF values were used to better identify possible SCNA events not detectable by the log2R signal. As in this case, focal regions were analyzed in their entirety without segmentation.

### Computation of allelic imbalance per segmented region

For each of the identified segments, an allelic imbalance value was computed using a methodology that extends our work in refs. ^27,55^. In detail, given a cfDNA sample and an identified SCNA segment *S*, the set of SNPs that are heterozygous in the cfDNA sample, contained in the segment and also present in the panel of controls were selected and used to compute a value representing the evidence of allelic imbalance for the segment *S* and another value representing an estimate of the ${\beta }_{S}$ value, which represents the proportion of local read depth signal that is not imputable to ctDNA^56^. The evidence of allelic imbalance was computed with the formula:

3 $$E\left({\rm{S}}\right)=\frac{\mathop{\sum }\nolimits_{1}^{k}W\left(d \,>\, D\right)}{k}$$

were *k* = 5 (by default), $d$ is the observed mAF distribution in the cfDNA sample, $D$ is a simulated mAF distribution generated sampling one time for each SNP in *S* from a normal distribution with mean and standard deviation obtained from the panel of controls, and *W* is a function returning 1 if the difference between ${d}$ and $D$ applying a Wilcoxon signed-rank test with significance cutoff of 1% is statistically significant, 0 otherwise.

The ${\beta }_{S}$ estimate was instead computed by comparing $d$ with simulated distributions mimicking different levels of *β* (representing different ctDNA levels) and searching for the most similar one. Formally:

4 $$\begin{array}{l}{\beta }_{S}=\min \left\{\beta \vee W\left(d \,>\, {D}_{\beta }\right)\right\}-\left(\min \left\{\beta \vee W\left(d \,>\, {D}_{\beta }\right)\right\}\right.\\\left.\qquad-\max \left\{\beta \vee W\left(d \,<\, {D}_{\beta }\right)\right\}\right)* P\end{array}$$

with

5 $$P=\frac{{median}(d-\min (d))}{\max (d)-\min (d)}\text{and}\,\beta \in \left\{0.01,0.02,\ldots ,0.99,1\right\}$$

and where $W(d \,>\, {D}_{\beta })$ is the Wilcoxon signed-rank statistics (significance cutoff of 1%) comparing $d$ and ${D}_{\beta }$.

### Assignment of copy number state

To assign a copy number state to each SCNA segment identified in a cfDNA sample, a computational approach combining allelic imbalance evidence and read-depth z-score thresholds is used. In detail, given a SCNA segment *S* identified in a cfDNA sample, the panel of controls *log2R table* was queried to obtain the set of captured genomic regions that are contained in the segment, denoted as ${R}_{S}$. Then, for each control sample, the median log2R of all ${R}_{S}$ genomic regions was computed resulting in a vector of reference log2R values for the segment *S*, denoted as ${\log 2R}_{S}$. The z-score for the segment *S* was then calculated as follows:

6 $${{zscore}}_{S}=\frac{({\log 2R}_{S}-{mean}({\log 2R}_{S}))}{{std}({\log 2R}_{S})}$$

A statistical *read-depth z-score* threshold ${Z}_{{thr}}$ (by default 2.58) was finally combined with an *allelic imbalance evidence* threshold ${E}_{{thr}}$ (by default 0.8) to assign a copy number state to each segment *S* in the following way:

7 $$SCN{A}_{s}=n\left\{\begin{array}{ll}imbalanced\,GAIN\,(iGAIN), & zscor{e}_{s} > {Z}_{thr}\wedge E(S) > {E}_{thr}\\ {monoallelic}\,{LOSS}\,({mLOSS}),&{zscor}{e}_{s} < -{Z}_{thr}\wedge {E}(S) > {E}_{thr}\\\qquad\,{allelic}\,{imbalance}\,(AI), &{zscor}{e}_{s}\in \,[-{Z}_{thr},{Z}_{thr}]\wedge {E}(S) > {E}_{thr}\\ \qquad\qquad\qquad\qquad\;\;{GAIN}, & {zscor}{e}_{s} > {Z}_{thr}\wedge E(S)\le {E}_{thr}\\ \qquad\qquad\qquad\qquad\;\,\;{LOSS}, &{zscor}{e}_{s} < -{Z}_{thr}\wedge E(S)\le {E}_{thr}\end{array}\,\right.$$

The state ${AI}$ is used to identify all segments that have evidence of allelic imbalance but for which there is no statistical evidence of SCNA from the read depth analysis. Of note, when the ctDNA level estimation (see below) for the sample is above 15% we can confidently assume that ${AI}$ events are ${LOH}$ events.

### ctDNA level and ploidy estimation

ctDNA detection in cfDNA plasma samples was assessed considering, for each sample, the presence of at least one segment having an allelic imbalance evidence value greater than ${E}_{{thr}}$. ctDNA level for a cfDNA sample was instead estimated considering the *β* values of all SCNA segments identified as ${mLOSS}$^56^$,$ which represent the set of mono-allelic deletions identified in the cfDNA sample. In detail, an integrated *β* value was calculated as a weighted mean of all peaks identified in the *β* segment values distribution (*β* was weighted by considering the magnitude of the peak). Then, as described in^56^, the ctDNA level was estimated as:

8 $${ctDNA}=1-\frac{\beta }{\left(2-\beta \right)}$$

For each cfDNA sample having evidence of tumor signal, a ploidy estimation was computed. In detail, samples segments were filtered for those with *β* equal to 1, indicating no presence of allelic imbalance. Clustering using dbscan^57^ of log2R values for these segments was then performed and the left most cluster was identified as the one representing the balanced copy number 2 (one copy per allele) and used as *shift* value to adjust the overall sample’ log2R distribution. More specifically, the adjustment was applied to all segments in order to center putative copy number neutral segments to zero:

9 $$\log 2R.{corrected}=\log 2R-{shift}$$

Of note, since ctDNA is typically low in cfDNA samples and ploidy values are extremely challenging to calculate at low ctDNA level, the ploidy adjustment was applied only when the ctDNA level estimation was greater than 15%. When ploidy adjustment was applied, a new ctDNA level estimation was calculated after the adjustment.

### In-silico benchmarking

To assess the theoretical detection/estimation limits of our approach, an *in-silico* cohort of synthetic samples was generated. To this end, synggen, a computational tool we recently developed for the fast generation of large-scale realistic and heterogeneous cancer sequencing synthetic datasets, was used^35^. To generate the *in-silico* cohort, profiles of germline SNPs and somatic allele-specific SCNA were collected and retrieved, respectively, from CEU individuals in the 1000 Genomes Project collection and from the TCGA dataset (cbioportal.org).

In detail, sequencing data (BAM files) of the control samples from the available cohort were provided in input to synggen using a specific execution mode that, from those files, extracts a series of statistical models that summarize platform specific data characteristics, such as the distribution of the read depth of coverage, the distributions of read and base qualities, and base-specific systematic errors. These models were then used, in conjunction with the collected SNPs and allele-specific SCNA profiles, to generate synthetic control and cfDNA samples at different levels of ctDNA and average depth of coverage.

More precisely, for a read depth of coverage of ~800x (representing the average coverage of the data we generated), 20 representative breast cancer samples for each of the following decreasing ctDNA level were generated [80%, 60%, 40%, 20%, 10%, 7.5%, 5%, 4%, 3%, 2%, 1%, 0.5%, 0.1%]. Then, for increasing read depth of coverage scenarios [1000x, 1500x, 2000x, 2500x], 20 representative breast cancer samples for each of the following decreasing ctDNA level were generated [5%, 4%, 3%, 2%, 1%, 0.5%, 0.1%]. In addition, 40 control samples were generated for each simulated depth of coverage scenario, half representing cfDNA samples with no tumor signal and half to generate the panels of controls. All allele-specific SCNA were included in the synthetic cfDNA data as clonal events.

The simulated cohort was used to assess the performance of our assay. In particular, we looked into the ability of our computational approach to identify presence of tumor signal in a synthetic cfDNA sample, namely a detection performance, and, if possible, into the estimation of the ctDNA level present in the sample analyzed. Detection performances were tested looking into the accuracy of identifying samples with positive ctDNA at each different simulated condition (coverage/ctDNA level) independently (*N* = 20).

### SNV calls

To detect somatic single nucleotide variants (SNVs) we applied ABEMUS^38^, a method we previously implemented and that is specifically designed for SNVs detection in cfDNA samples. ABEMUS was run using our control samples to create the ABEMUS reference model, which captures platform specific characteristics that are used by the tool to improve precision of SNVs calling. ABEMUS analyses were then performed using the standard computational workflow across all cfDNA samples considering only the exonic regions that are captured by the panel (i.e. we excluded all regions capturing assay SNPs). Considering that we had no matched control samples for our cfDNA samples, we then implemented an ad-hoc post-processing strategy to reduce the number of false positives. First, we annotated all identified position using the SNP Nexus web server. Exploiting the sequential samples, we then excluded all identified SNVs that were annotated in the dbSNP database, had a MAF > 0.01 in gnomAD and had in 2/3 of the cfDNA sequential samples an AF > 0.2. From the remaining calls, we then excluded the ones that were annotated in the dbSNP database, had a MAF > 0 in gnomAD and had in all the sequential cfDNA samples an AF > 0.4. Finally, we retained only the SNV calls supported by a number of alternative reads >1 and annotated for the presence in the COSMIC database^58^ or in breast cancer datasets available from the cBioPortal. The clonality of single nucleotide variants (SNVs) was determined by calculating the ratio of the SNV’s allele frequency to the sample’s ctDNA level. This calculation assumes the presence of a mono-allelic mutation and includes a correction for mLOSS. If the resulting ratio exceeds 1, it is normalized to 1. Values above 0.75 are considered associated to clonal SNVs.

### Statistical analyses

Correlation of ctDNA levels’ estimations was performed using Pearson correlation statistics with significance level set at 5%. Univariate overall survival and progression-free survival analyses were performed using the Kaplan-Meier estimator (log-rank test).

## Supplementary information

Supplementary Data

Supplementary Information

## Data Availability

Processed data of cfDNA samples are available upon request. The BAM files for the control samples have been deposited in the EGA archive under accession number EGAD50000001167.

## Code availability

eSENSES method is available as R script at https://github.com/cibiobcg/eSENSES.
