# Supplementary material for: Enabling sensitive and precise detection of ctDNA through somatic copy number aberrations in breast cancer
Source: NPJ Breast Cancer. 2025 Mar 8;11:25. doi: 10.1038/s41523-025-00739-6 (PMC11890748; doi:10.1038/s41523-025-00739-6)
Supplement: Supplementary file 2 — Supplementary Information [file 41523_2025_739_MOESM2_ESM.pdf]

Supplementary Data

Supplementary Data 1: Genes with coding regions included in the panel

Supplementary Data 2: Details of eSENSES assay probes

Supplementary Data 3: Patients' characteristics

Supplementary Data 4: ctDNA detection and estimation results for patients' samples

Supplementary Data 5: Patients' clinical data

Supplementary Data 6: SCNAs detected in our patients' cfDNA samples

Supplementary Data 7: Non-synonymous SNVs detected in our patients' cfDNA samples

Supplementary Data 8: Frequencies of PIK3CA, TP53 and ESR1 non-synonymous SNVs in our study and other large genomic studies

Supplementary Figures

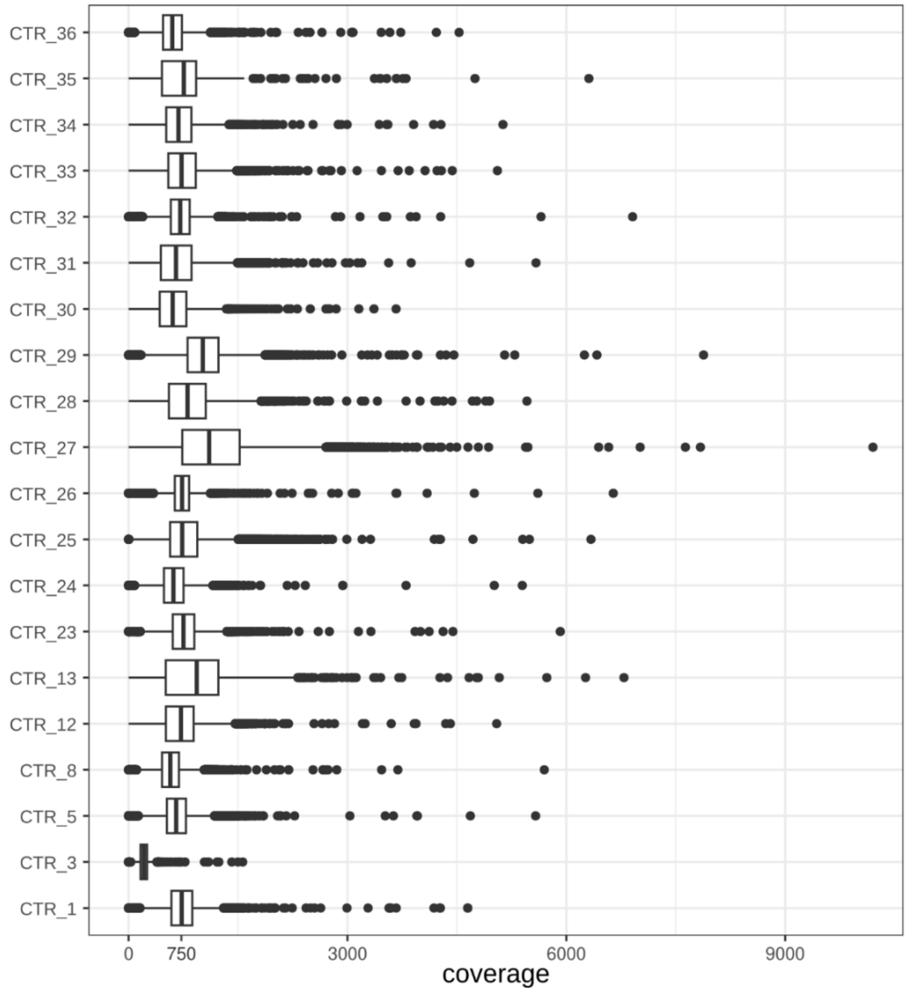

Supplementary Figure 1: Processed control samples' distributions of read depth coverage.

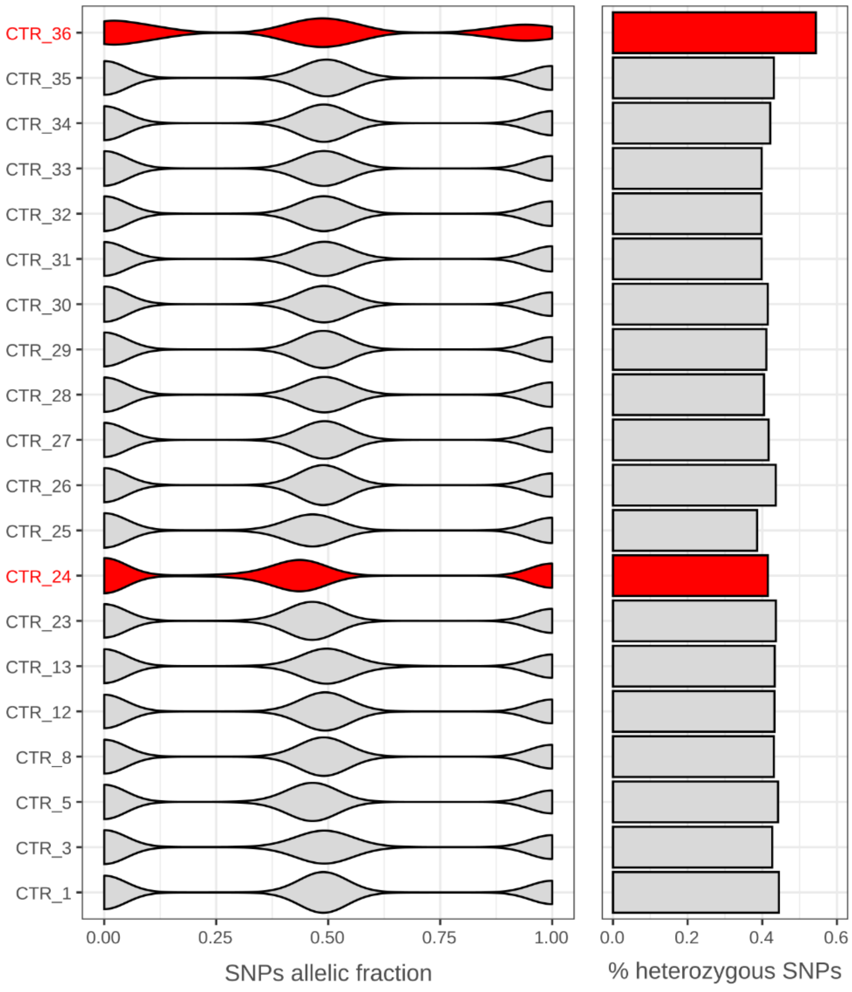

Supplementary Figure 2: Processed control samples' distributions of allelic fractions with reported percentage of heterozygous SNPs (allelic fraction between 0.1 and 0.9) among total SNPs per patient. Patients highlighted in red were removed due to the not standard distribution of allelic fraction.

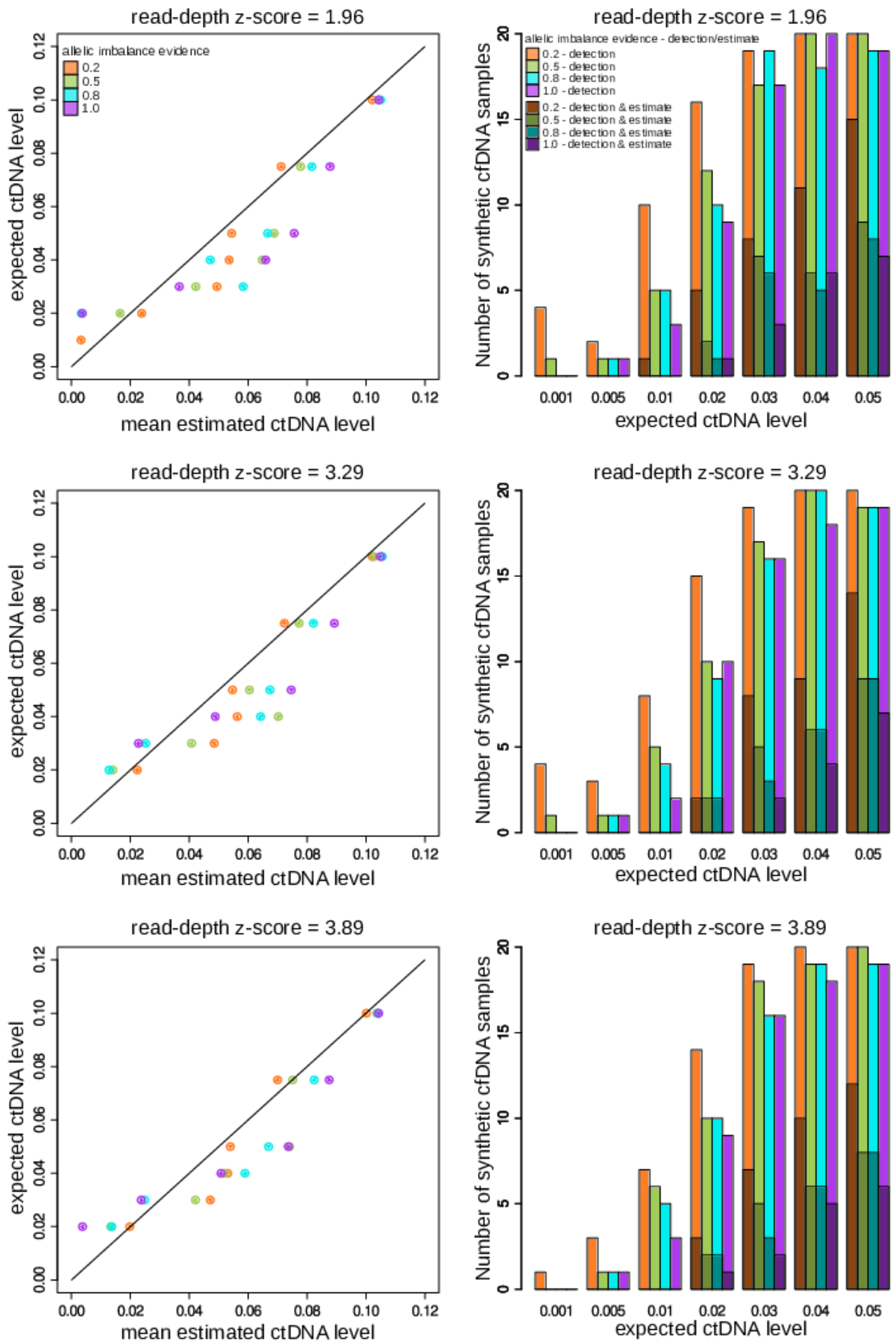

Supplementary Figure 3: (Left) Concordance between the mean estimated ctDNA level, calculated across the results obtained for the simulated cfDNA samples, and the expected ctDNA level. Results are shown for increasing read-depth z-score parameter and increasing allelic imbalance evidence parameter' values. (Right) Fraction of samples with ctDNA detection and ctDNA level estimation for expected ctDNA levels up to 5%. Results are shown for increasing read-depth z-score parameter and increasing allelic imbalance evidence parameter' values

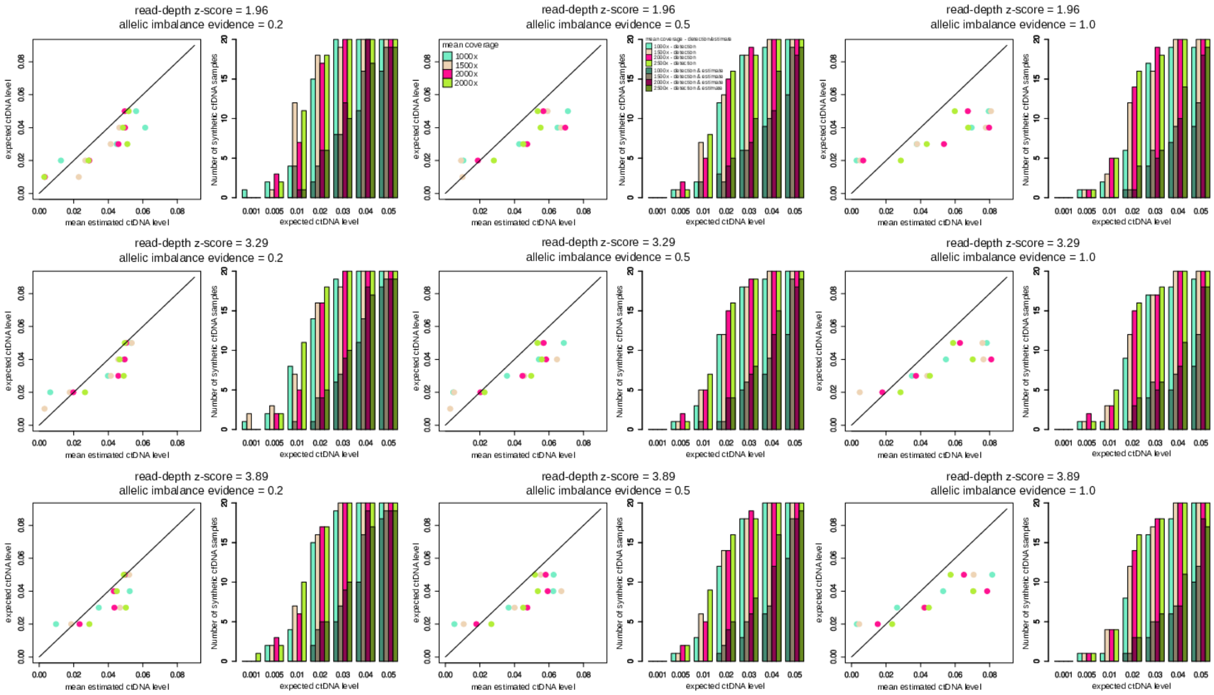

Supplementary Figure 4: Scatterplots show the concordance between the mean estimated ctDNA level, calculated across the results obtained for the simulated cfDNA samples, and the expected ctDNA level. Results are shown for increasing read-depth z-score parameter and increasing allelic imbalance evidence. Barplots show the fraction of samples with ctDNA detection and ctDNA level estimation for expected ctDNA levels up to 5%. Results are shown for increasing read-depth z-score parameter and increasing allelic imbalance evidence.

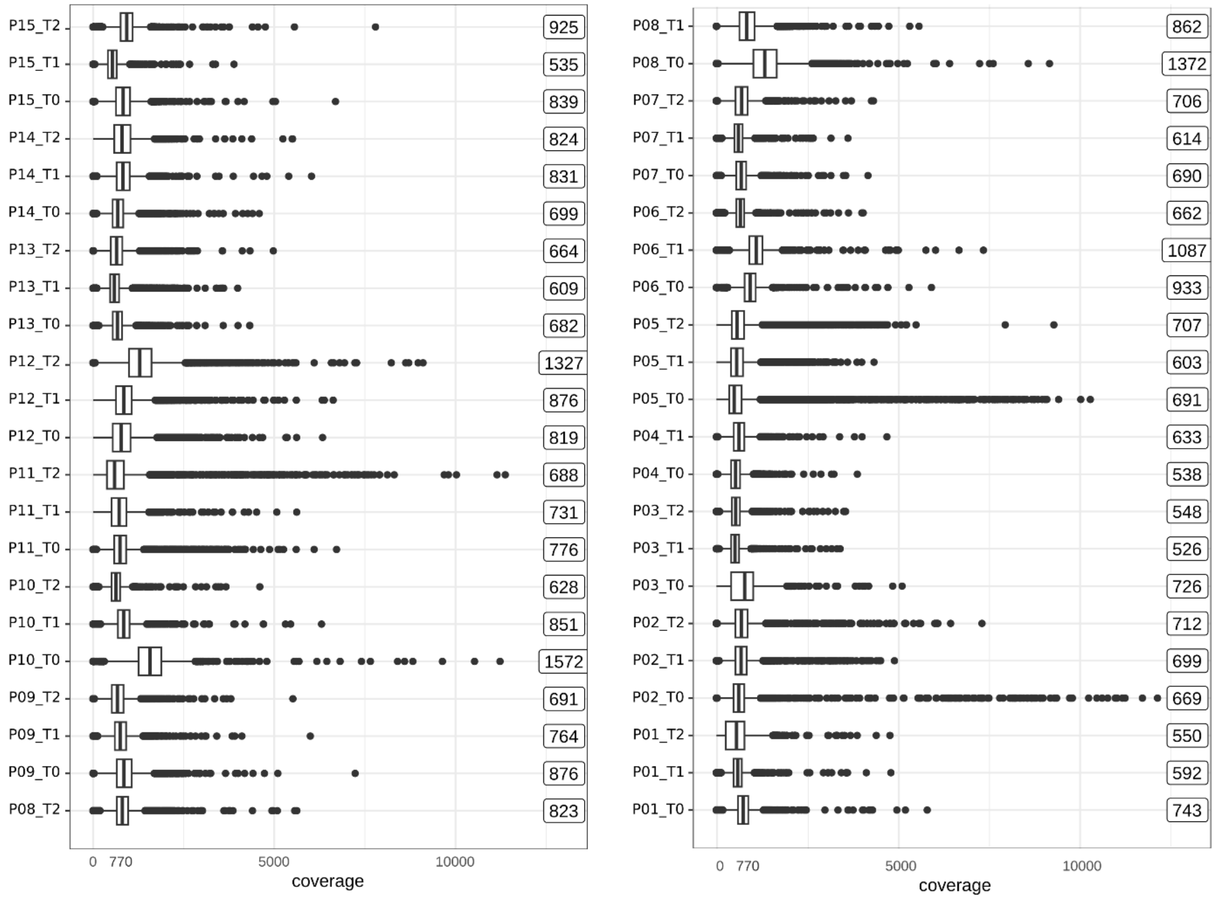

Supplementary Figure 5: Processed patients' cfDNA samples' distributions of read depth coverage.

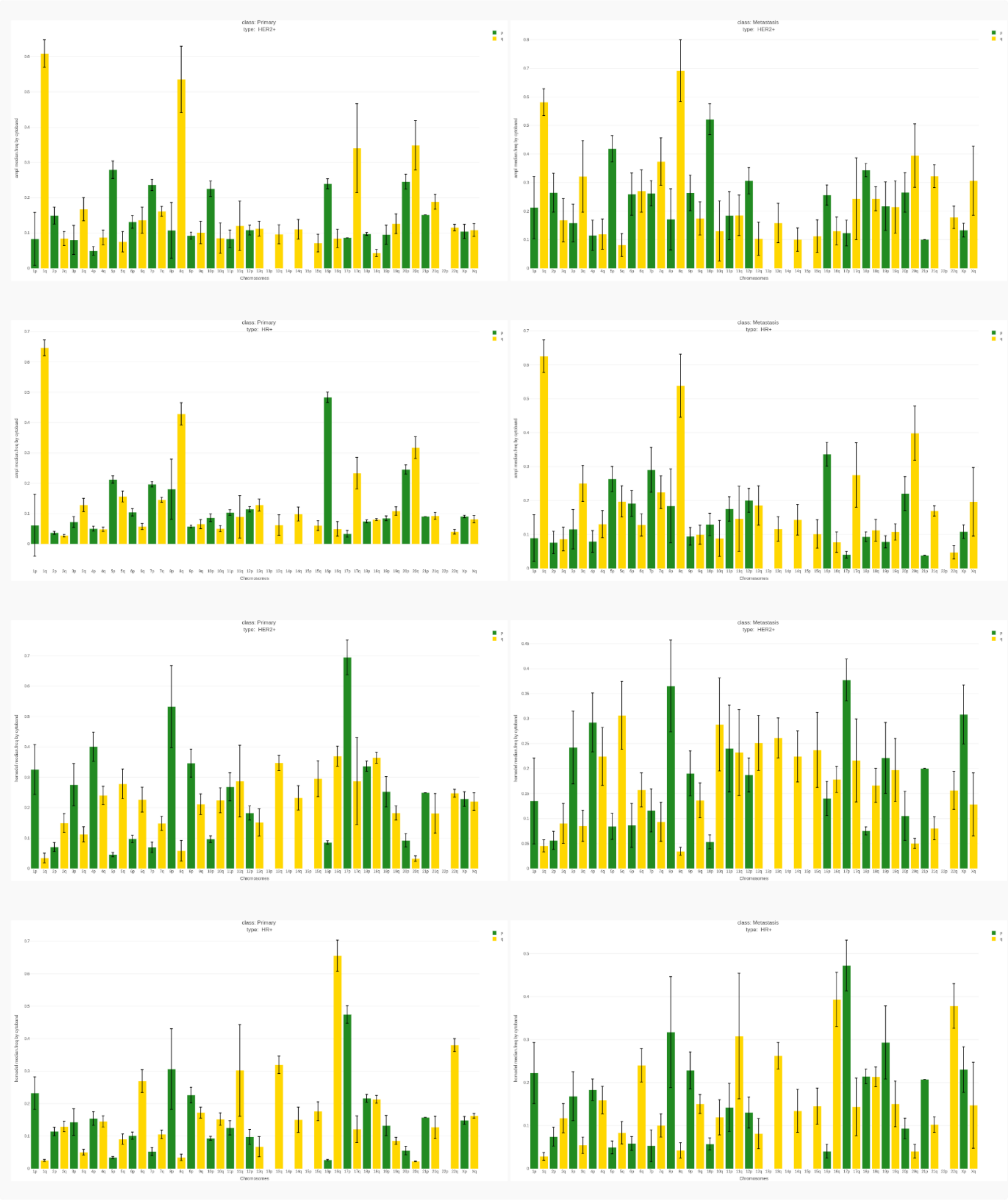

Supplementary Figure 6: Arm-level SCNA frequencies retrieved across multiple large scale genomic studies obtained using BroadBand (bcglab.cibio.unitn.it/broadband), a web-based resource we developed for exploring genomic data selected from a comprehensive array of breast cancer studies containing information on SNVs and SCNAs.

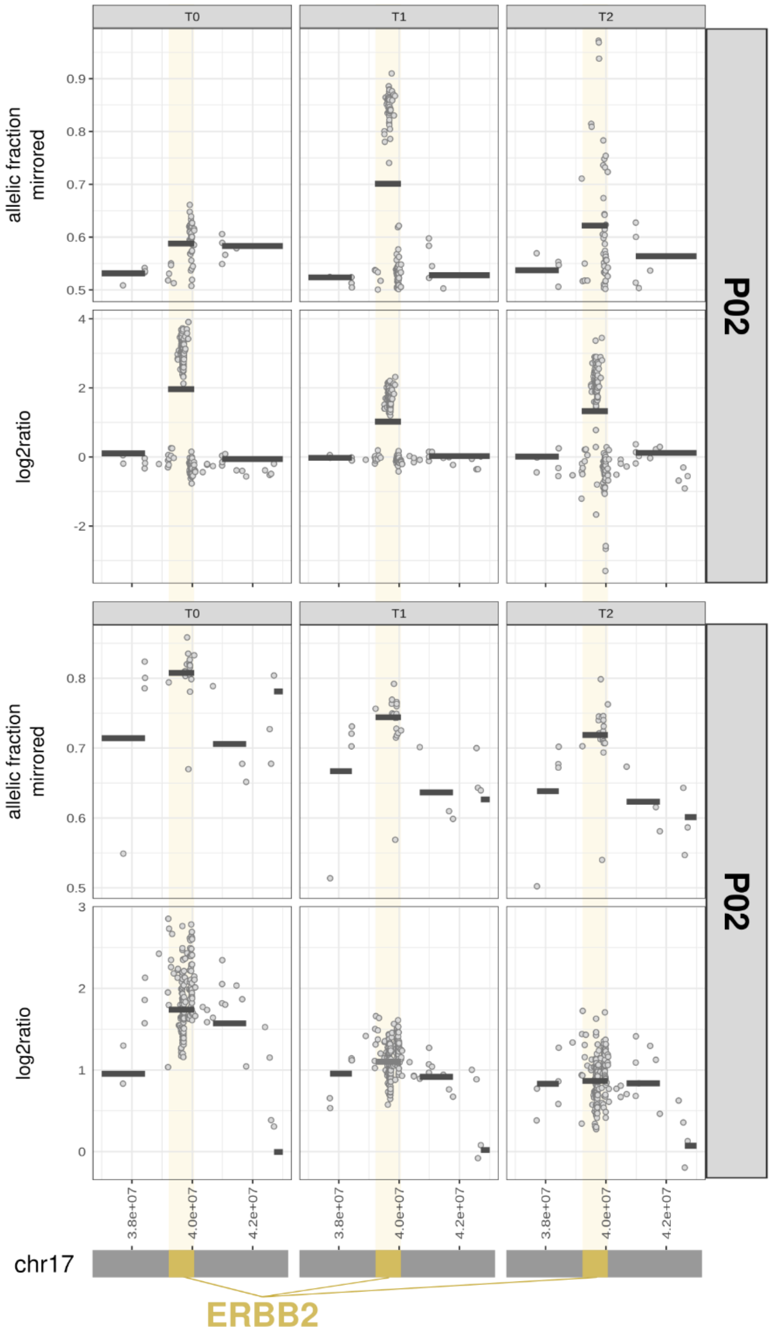

Supplementary Figure 7: Log2 ratio and mirrored allelic fraction signals at each time point of the two HER2+ patients showing detected focal somatic copy number segments corresponding to the genomic region of *ERBB2* gene.

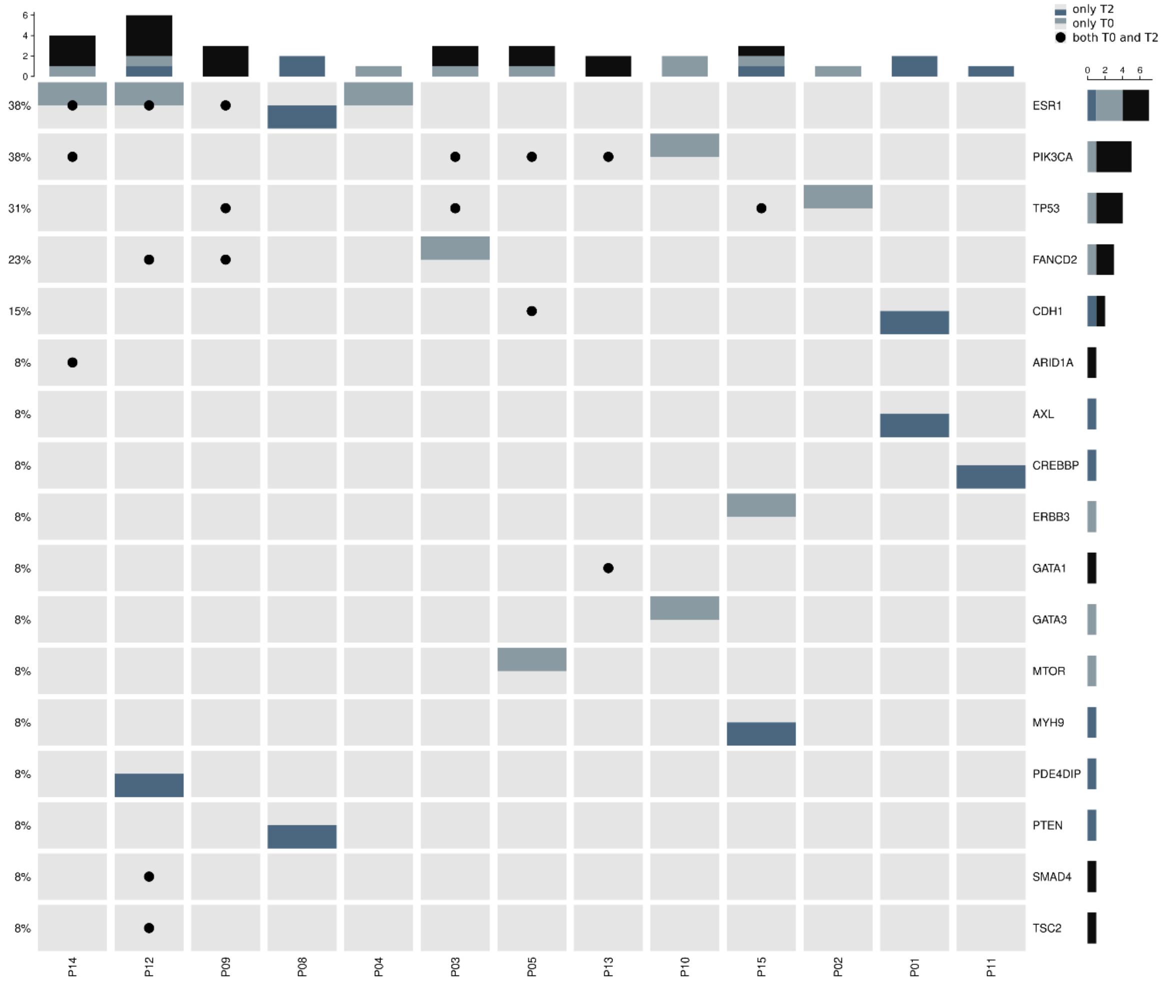

Supplementary Figure 8: Oncoplot of non-synonymous SNV detected across patients' cfDNA samples at T0 and T2.

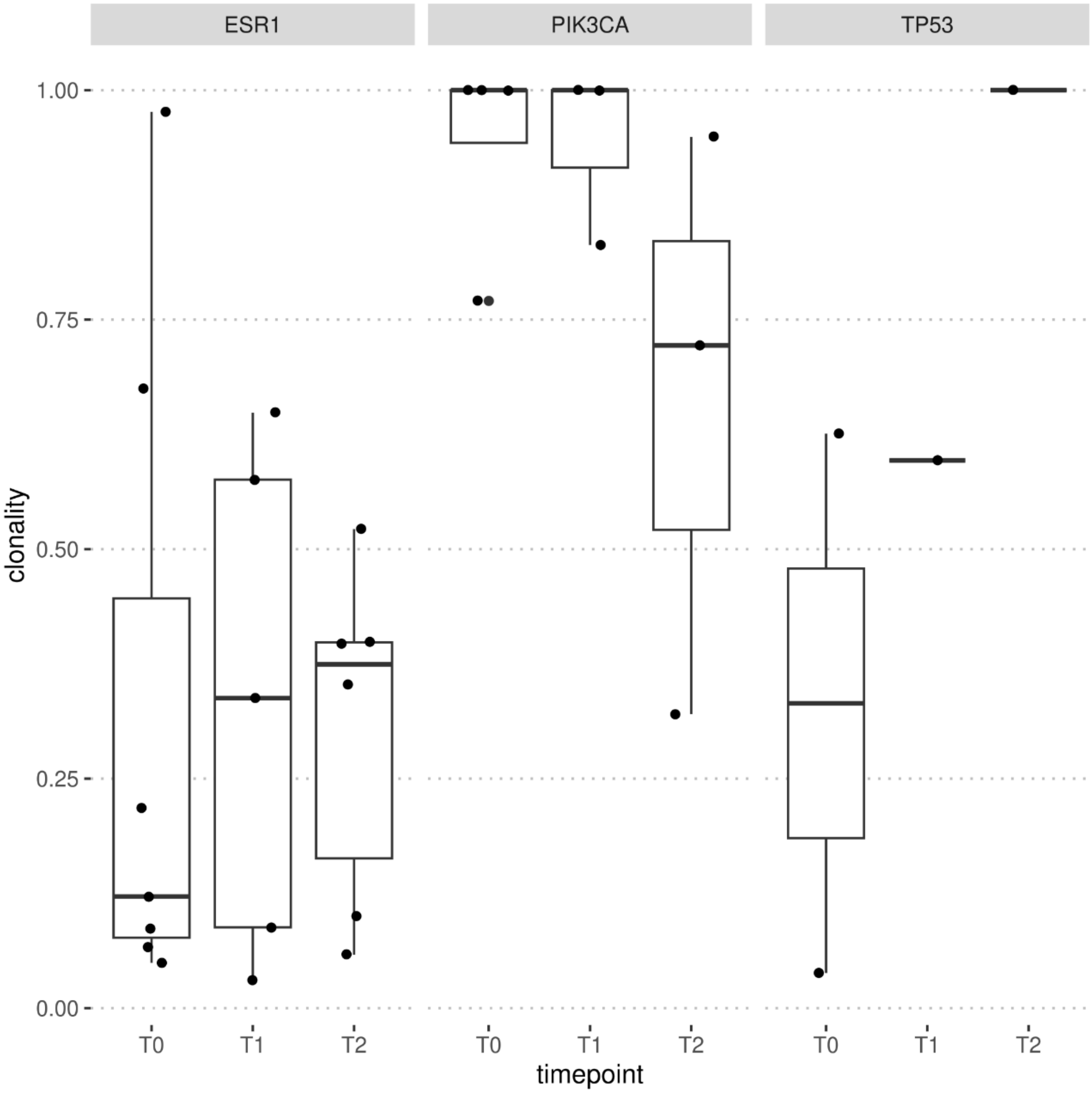

Supplementary Figure 9: Clonality of damaging/deleterious SNVs across the most frequently mutated genes. Values above 0.75 are considered associated to clonal SNVs.

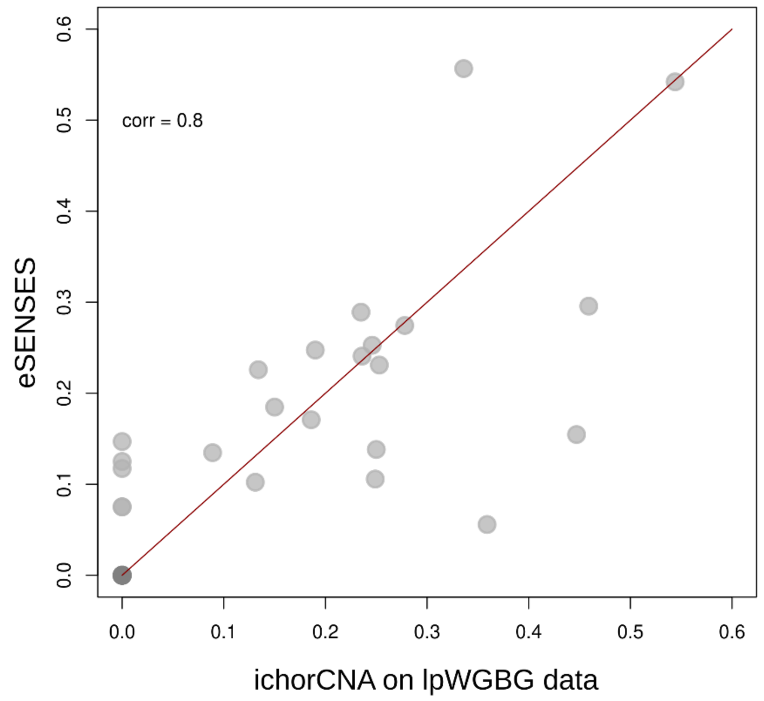

Supplementary Figure 10: Concordance of eSENSES ctDNA level estimations with estimations obtained with ichorCNA (*default* parameters) from lpWGBS samples available for the same patients at the same time points.
